# Perceptions of the Impact of Comorbidity on the Bowel Cancer Screening Programme: Qualitative Study With Bowel Screening Participants and Staff

**DOI:** 10.1111/hex.14126

**Published:** 2024-07-02

**Authors:** Delyth Price, Katherine Brain, Sunil Dolwani, Adrian Edwards, Kerenza Hood, Stephanie Smits

**Affiliations:** ^1^ PRIME Centre Wales Cardiff University Cardiff UK; ^2^ Division of Population Medicine Cardiff University Cardiff UK; ^3^ College of Biomedical & Life Sciences Cardiff University Cardiff UK

**Keywords:** bowel screening, comorbidity, patient experiences, qualitative, screening experiences

## Abstract

**Introduction:**

The impact of multiple health conditions on bowel cancer screening is currently unknown. We explored the impact of multiple health conditions on bowel cancer screening perceptions, experience and clinical management decisions following a positive stool test.

**Methods:**

Semi‐structured qualitative interviews were conducted remotely with Bowel Screening Wales staff (*n* = 16) stratified by regional location and role and with screening participants (*n* = 19) stratified by age, gender and comorbidity. Interview topics were guided by the Common‐Sense Model.

**Results:**

Screening participants, regardless of comorbidity status, placed great emphasis on the importance of early detection of cancer and completing the bowel screening process. Screening staff emphasised comorbidities in the clinical decision‐making process; however, screening participants had low awareness of the impact that comorbidities can have on bowel screening. Participants describe how the presence of multiple health conditions can mask potential bowel symptoms and influence beliefs about follow‐up.

**Conclusion:**

Bowel screening staff try to individualise the service to meet participant needs. The potential mismatch in screening staff and participant awareness and expectations of the bowel screening and diagnostic process needs to be addressed. Clearer and more regular communication with screening participants could support the screening process, particularly for those with significant coexisting health conditions or facing time delays. The possible masking effects and misattribution of symptoms because of comorbidities highlight an opportunity for education and raising awareness for screening participants and a potential area of focus for discussions in clinical consultations and staff training.

**Patient and Public Contribution:**

Project funding included costs for patients and public contributors to be compensated for their contributions to the project, in line with current standards. A patient and public contributor was involved in the design of the study, including protocol development, and the interpretation of key findings and implications for patients, which are subsequently reflected within the manuscript.

## 1 Introduction

Bowel screening in the United Kingdom has many stages, starting with uptake via a home‐based stool test (faecal immunochemical test, FIT), and if positive, consultation with a specialist screening practitioner (SSP), follow‐up of abnormal results, diagnostic and, if appropriate, surgical procedures, with subsequent surveillance and rescreening. The presence of coexisting health conditions in screening participants may impact how they engage with and experience the various stages of bowel screening, as well as the potential benefit they may receive in terms of improvement in outcomes. Comorbidity is the presence of one, and multimorbidity is the presence of two or more concurrent health conditions [1, 2]. Based on age, estimates suggest multimorbidity rates of 40%–60% among people eligible for bowel cancer screening [1, 3]. Multimorbidity is therefore an important factor in the screening programme. Multimorbidity can impact clinical factors, including procedural risk estimates, the use of alternative tests and the assessment of physical limitations [4]. From a health service perspective, considering co‐ or multimorbidity when designing services and implementing staff training is important due to the prevalence of health conditions in service users [5]. From a policy perspective, ensuring that screening participants adhere to appropriate follow‐up and diagnostic procedures is needed for the service to be appropriately delivered and cost‐effective. Ensuring screening‐eligible individuals are aware of risks/benefits (including potential implications of multimorbidity) is also important from an informed decision‐making perspective.

Multimorbidity, associated life expectancy and quality of life [6, 7, 8] are important considerations for healthcare professionals advising screening participants as they progress through bowel cancer screening. For screening participants, co‐ or multimorbidity may influence perceived ability to complete screening follow‐up, diagnostic and surgical procedures and treatment, as well as the perceived risk of developing bowel cancer. People may focus on one health condition at a time [9], and consequently, for those with multimorbidity, bowel cancer screening experience, completion and outcomes may be influenced by existing health conditions, which predominate in terms of health concerns, to the detriment of bowel cancer screening uptake/completion.

Screening experiences and referral pathway timelines need exploration as complexities associated with multimorbidity could lead to diagnostic pathway delays [10, 11]. Whilst recent research has explored the impact that health conditions have on bowel cancer diagnosis via the symptomatic or emergency presentation route [12, 13], there remain evidence gaps for the bowel screening route. Research that has explored bowel screening and comorbidity has focussed on screening uptake [13, 14] and not downstream processes, which take place once screening has commenced and a positive stool test is received, such as follow‐up, diagnostic testing and treatment.

The Common Sense Model of Illness Self‐Regulation and Health [15] can be used to understand how perceptions of health conditions may influence experiences and decisions within the screening programme. This states that a sense model guides people to make decisions about their physical, emotional and social states of well‐being [15]. Illness representations are beliefs and cognitions through which people understand and make sense of illness or health threats. These play an important role in generating emotional experiences and influencing subsequent behaviour. The model identifies five key components of illness representation: (1) *identity* is the label the person uses to describe the illness, symptoms or health threat; (2) beliefs about the *cause*; (3) *timeline* is the expected duration; (4) *consequences* are the expected effects and outcomes; and (5) *control/cure* refers to the responsiveness of the symptoms, illness or health threat to treatment or self‐management (see Table 2 for more detail).

Applied to the current context, the Common Sense Model states that information about a health threat, such as receiving a positive stool test, activates illness representations across the five dimensions [15] for screening participants. For bowel screening staff, when considering the impact of screening participant health conditions, illness representations may drive clinical management and referral decisions.

To address the current gaps in knowledge, the aim of this study was to understand the impact of health conditions on experiences, perceptions and clinical management decisions following a positive stool test. We carried out qualitative interviews with health professionals working within Bowel Screening Wales (BSW) and screening participants with varying levels of (or no) comorbidity.

## 2 Methods

### 2.1 Study Design

A qualitative research approach was adopted, with narrative inquiry used to gain in‐depth understanding on how health conditions and other factors impact experiences and decision‐making for staff and screening participants within the bowel screening programme.

### 2.2 Study Setting

There are 13 local assessment centres in the Welsh bowel screening programme (BSW) across the seven Health Boards in Wales (Aneurin Bevan University, Betsi Cadwaladr University, Cardiff and Vale University, Cwm Taf Morgannwg University, Howell Dda University, Powys Teaching and Swansea Bay University).

During data collection, BSW invited people aged 54–74 to participate in the screening. The programme subsequently expanded from October 2023 to invite people aged 51–74 [16].

### 2.3 Sampling and Participants

#### 2.3.1 Screening Participants

In the Welsh bowel screening programme (BSW), people who receive a positive stool test have a telephone consultation with a SSP and are sent an information pack following this consultation, before having a follow‐up appointment (typically, a colonoscopy). BSW posted our study materials (consent form, information sheet and health questionnaire) along with this routinely posted information pack to 130 people who underwent screening and received a positive stool test (10 postal packs from each of the 13 local assessment centres). Convenience sampling was used to recruit screening participants with a range of health conditions (including no health conditions) who were willing to take part in an interview. Participants posted their signed consent form and health questionnaire to the research team in Cardiff University, who contacted them by telephone to discuss the study and arrange an interview. Interviews were arranged to take place after screening participants' follow‐up appointments to enable exploration of their experiences of follow‐up. All willing participants were invited to take part in an interview.

#### 2.3.2 Screening Staff

BSW staff, including SSPs and screening colonoscopists, were identified via existing professional contacts and invited via email to take part in an interview. Screening staff sent their signed consent form via email or post and were contacted via email or telephone to discuss the study and arrange an interview. Convenience sampling was used, with data collection ending when the team were satisfied with the level of representation from job role and location.

### 2.4 Data Collection

Screening participants' self‐reported health conditions were captured using a validated, self‐administered comorbidity questionnaire [17]. The questionnaire consists of 13 conditions, with three questions asked in relation to each condition: (1) Do you have problem? (2) Do you receive treatment for it? (3) Does it limit your activities? Answers to these questions were used to gain insight into participants' health status and the impact of any health conditions on their lives, for further exploration during interviews. The Welsh Index of Multiple Deprivation (WIMD) is the official measure of deprivation in Wales. These scores were calculated using screening participants' postcodes to provide insight into the levels of deprivation amongst participants.

Semi‐structured interviews were conducted remotely, audio‐recorded and transcribed verbatim. Individual topic guides (see Appendices S1 and S2) were developed for the screening participant and screening staff interviews, guided by the Common Sense Model [15]. Topics explored during screening participant interviews included perceptions of health and comorbidities, perceptions and experiences of bowel screening and how comorbidities impact these. Topics explored with screening staff included the impact of comorbidities on bowel screening and clinical decision‐making for participants with comorbidities. Improvements to bowel screening were explored in interviews with both staff and screening participants.

### 2.5 Data Analysis

Interviews were transcribed verbatim by an approved third party and checked for accuracy by the study team. Anonymised transcripts were analysed inductively, guided by thematic analysis [18, 19] and supported by qualitative data analysis software (NVivo 12 and 1.7.1). Interviews were analysed by two experienced qualitative researchers (screening staff by S.S. and screening participants by D.P.). Authors initially familiarised themselves with the data and generated initial codes. Authors dual‐coded 20% of each other's interview transcripts and met to discuss coding frameworks and subsequently develop themes. Themes were then mapped to elements within the Common Sense Model (identity, cause, timeline, consequences and control/cute), which determined their significance for reporting. A patient and public contributor reviewed key findings from screening participant interviews and provided feedback on our interpretations.

## 3 Results

Between May and October 2022, 16 staff interviews were conducted with an average duration of 29 min (range 21–40 min). Between October 2022 and April 2023, 19 screening participant interviews were conducted with an average duration of 32 min (range 17–52 min). Consent forms were received from 24 screening participants; of these, two participants were uncontactable, and three participants declined when contacted before an interview being arranged.

Screening staff represented all Health Boards within Wales and all roles within the screening programme. Screening participants were predominantly from North and West Wales. The sample of screening participants included 10 men and nine women, with an average age of 65 (range 58–74). WIMD scores ranged from 2 to 5, with more screening participants living in areas of lower deprivation. The most common conditions reported by screening participants were high blood pressure, diabetes, back pain and anaemia or other blood disease (see Table 1 and File S1). Quotes in Section 3 are flagged as either being from the screening participants (SPart) or bowel screening health professionals (HProf).

**Table 1:** Bowel screening participant and screening staff characteristics.

	Screening participant	Health professional
Role	n/a	
Specialist screening practitioner		6
Screening colonoscopist		9
Clinical quality assurance coordinator		1
Gender		
Female	9	8
Male	10	8
Health conditions		n/a
Heart disease	3	
High blood pressure	8	
Lung disease	1	
Diabetes	5	
Ulcer or stomach disease	1	
Kidney disease	1	
Liver disease	3	
Anaemia or other blood disease	4	
Cancer	3	
Depression	3	
Osteoarthritis or degenerative arthritis	3	
Back pain	4	
Rheumatoid arthritis	3	
Welsh Index of Multiple Deprivation score		n/a
1 (*most deprived*)	0	
2	3	
3	4	
4	5	
5 (*least deprived*)	7	

Results were mapped to the five illness representations in the Common Sense Model, displayed in Table 2 below.

**Table 2:** Application of the Common Sense Model to the current study.

Common Sense Model theme	CSM theme description	CSM theme application to the current study
Identity	The label the person uses to describe the illness, symptoms or health threat viewed as being part of the disorder.	Descriptions and perceptions of the health conditions, and perceptions of bowel cancer.
Cause	Beliefs about what caused the symptoms or illness.	Beliefs about the cause of symptoms, the cause of the positive stool test and beliefs about screening.
Timeline	The expected duration of the symptoms or illness.	Expected duration of the screening journey and chronic nature of health conditions.
Consequences	Expected effects and outcomes of the illness.	Expected effects and outcomes of undergoing screening, and perceived impacts of health conditions.
Control/cure	Responsiveness of the symptoms or illness to treatment or self‐management.	Beliefs about bowel screening and control in relation to health conditions, including coping with follow‐up procedures and self‐management in the context of health conditions (e.g., medications).

### 3.1 Identity (*Descriptions and Perceptions of the Health Conditions, and Perceptions of Bowel Cancer*)

Health conditions were described by screening staff as being very prevalent amongst screening participants, with this expected because of the screening eligibility age.

Well, you know, bowel screening is relevant to an age group erm where multi‐morbidity is common, so we, we see, yeah, obviously get a lot of erm patients who have other health problems and it does give us some sort of conflict in terms of whether it's appropriate that we investigate them further.

(HProf8)

Specific health conditions and patient groups were mentioned by screening staff when reflecting on different challenges in the screening programme. Conditions were wide‐ranging and included neurological problems, cardiovascular issues, cancer diagnoses and being on a palliative care pathway. Central to these reflections was the individual nature of the impact of conditions and the importance of not taking the comorbidity label at face value.

People don't fit into boxes you know, it's nice to have nice neat boxes, we all like tick boxes but people don't always fit in them, you know, so, we have to be aware of that isn't it, and it is about adapting our work.

(HProf17)

Linked with specific conditions were examples of high‐quality care and steps taken to try to minimise the burden of health conditions. For example, screening participants with diabetes would be brought in early, and hoists or additional staff were used for those with mobility issues. However, when discussing the impact of health conditions, screening participants were generally not aware of, or did not mention, specific mitigations put in place to manage their conditions. Many did not consider their conditions in the context of bowel screening at all. Screening participants did discuss specific concerns related to their own personal circumstances and identity, but these were not necessarily related to their comorbidities. For example, practicalities around the timing and location of appointments, concerns of discomfort during the colonoscopy and difficulty with bowel preparation were mentioned.

Um, well the only thing I was worried about was making a mess in the car going on the morning, because I … had to be there for nine or half past nine in the morning, which I had to have my second lot of powder about six o'clock in the morning. And then there was the drive from here to the hospital, and I was just afraid of having an accident on the way to, you know, having an accident in the car.

(SPart22)

These concerns were sometimes heightened for those with specific health conditions. For example, one participant with an inflammatory bowel disease described specific issues with taking part in bowel screening and concerns about a colonoscopy worsening their condition and symptoms.

### 3.2 Cause (*Beliefs About the Cause of Symptoms, the Cause of the Positive Stool Test and Beliefs About Screening*)

Some screening participants expressed feeling initial concern and worry following their positive stool test, and a desire to find out about and deal with any potential issues as soon as possible. For some, their health conditions masked potential symptoms, meaning the positive stool test was not unexpected. This also influenced beliefs about whether a follow‐up appointment was needed, and whether to have a colonoscopy.

Er, so I don't think I need to go … you know, have a camera job if there is blood, because according to what I think, and the nurses think … er, the nurse I'm dealing with for my colitis, er, it's more than likely that is causing the bleeding.

(SPart17)

When screening staff have discussions with screening participants about clinical decisions or recommendations, communication skills are drawn upon.

… kind of take account of the co‐morbidity, um, to inform the risk benefit, um, conversation with the patient.

(HP12)

These conversations and skills are used by screening staff to help increase screening participants' understanding of the causal influences of the clinical recommendations and decisions. This was described as particularly important when consulting with screening participants with health conditions to help increase understanding of the potential impacts of their health conditions on screening. The concept of shared decision‐making was mentioned by screening staff with a desire to have informed and empowered screening participants as a common theme.

### 3.3 Timeline (*Expected Duration of the Screening Journey and Chronic Nature of Health Conditions*)

Screening participants reflected on the difficulty of the long wait (some described waiting several months) between the positive stool test and follow‐up appointment, with waits associated with interim management of comorbid health issues, leading to heightened worry and stress. For those with more complex screening journeys, for example, requiring multiple colonoscopies, the additional waiting time between appointments further heightens stress and worry. Screening participants suggested that increased communication between appointments could mitigate this worry, by providing reassurance that they have not been forgotten. Many screening participants, however, understood the pressures on the NHS and found the waiting time between appointments to be acceptable, particularly in comparison to waiting times for other health services.

Maybe they say, after maybe 2 months say … ‘to let you know we've not forgotten you … your appointments are all set’ and this, that and the other and things like that just to let them know. Because in that waiting time for your first appointment it's probably … you know, the worry's there.

(SPart14)

Screening staff take time to build a clinical picture of screening participants' current health. This can involve using the clinical portal, reviewing guidelines and engaging with panels, such as multidisciplinary teams (MDTs) or CQAs (clinical quality assurance) and other clinical care teams.

It may be that I feel somebody probably would struggle and they [GP] might say to me ‘well no, actually they'd be okay’ and vice versa.

(HProf5)

In some cases, a face‐to‐face consultation is arranged in addition to the initial telephone preassessment to have further assessments. Timelines sometimes influence clinical decision‐making; for example, proximity to the screening upper age limit, life expectancy and individual impacts of health conditions. Potential future cause of death, and whether this would be likely to be attributed to the existing comorbidity(s) or (potential) bowel cancer, was also considered in some cases.

Sometimes it's so clear that these participants are not … never going to be fit, you know? They're never going to improve their condition. It is what it is, and it's only going to deteriorate.

(HProf7)

### 3.4 Consequences (*Expected Effects and Outcomes of Undergoing Screening, and Perceived Impacts of Health Conditions*)

When building a clinical picture for screening participants with health conditions, mobility (e.g., ability to get on a consultation table or hold a position), general fitness levels, life expectancy and tolerance for subsequent procedures are often considered by screening staff. The latter is based on perceived avoidance of harm if diagnostic work‐up and further treatment cannot be implemented. When discussing the consequences of screening, care home residents were the most frequently mentioned patient group by screening staff, with consent issues, awareness of next steps and appropriateness of completing the stool test discussed. Capacity issues, including cognitive and/or physical impairments, were also mentioned more generally in relation to follow‐up discussions. People with a mental health diagnosis and people in prisons were also specifically mentioned, with additional barriers for screening highlighted.

You know, if somebody's really not fit … you know, I had one lady who had multiple sclerosis and was bed‐bound. I mean, if we found a cancer, what are we going to do for her?

(HProf3)

When you offer a FIT and it's positive, what are the consequences then for that patient? Particularly should they need surgery? There's so much best interests decisions. So I think that group is, you know … whenever I've had to assess anybody with learning disabilities that's always taken me a lot of time and a lot of people to speak to, and erm you know to do the right thing for the patient really.

(HProf5)

Many screening participants experienced extreme difficulty in completing bowel preparation, reporting this was the worst part of the screening process and more unpleasant than the colonoscopy itself. Some even suggested that it would put them off going through bowel screening in future. This unacceptability of the bowel preparation was reflected across almost all screening participants interviewed, regardless of their health status or other demographics. Suggestions for bowel preparation improvements included taking a tablet instead of liquid, improving the taste of the liquid and more prior information and warning about the difficulty and unpleasantness. Screening staff reflected that bowel preparation influenced clinical decision‐making when consulting with screening participants with comorbidities, with the ability to complete and tolerate the bowel preparation used as an indicator for coping with the demands of follow‐up and treatment. Face‐to‐face consultations following the telephone consultation were utilised to further assess those with health conditions and their suitability for tolerating bowel preparation and subsequent procedures.

I didn't think it would be as awful as it was because that was the worst part about it, it's awful … you know, I'd have another colonoscopy tomorrow but drinking the drink was just awful. I knew it wasn't going to be pleasant but it was far more unpleasant that I ever thought it would be and I really struggled to get the drink down.

(SPart02)

And I think, I think it's often when we meet these patients, face to face, that's often the biggest as in whether they cope with the, with the bowel prep, more than anything else. And then you've also got the … should we find anything, what, what would we do? You know, if there was a, a tumour somewhere … would we then, you know, would this patient be suitable for any aggressive suitable treatment, you know?

(HProf11)

The opt‐in nature of bowel screening led to a sense of clinical obligation amongst screening staff to provide a good service, and for screening participants led to a perceived expectation of follow‐up. Screening staff reported that following the receipt of a positive stool test, screening participants put importance on knowing whether they have bowel cancer, this often being irrespective of the presence of health conditions.

Erm, er, I always knew where [the screening journey] would end up if the test was positive, that [a colonoscopy] would be recommended. And, er, you know I can't really see the point of going through all the, er, all the pre‐testing stuff … with you know the poo and that if you're not prepared to go ahead and have the, have the colonoscopy.

(SPart06)

Overall, many screening participants stated that an alternative and less invasive test was needed before having a colonoscopy (or in‐between screening). Reasons for this included perceptions that their comorbidities may increase the likelihood of blood in the stool or increase discomfort during a colonoscopy, or more generally, the invasiveness or discomfort of having a colonoscopy. Screening participants' desire for alternative tests was also evident in health professional interviews, with some screening staff reflecting on recent instances of screening participants requesting a computed tomography colonography (CTC). This was attributed to recent changes to information packs, which now include information being provided on CTCs. It described how screening participants believed that they could choose between a colonoscopy and CTC for their follow‐up procedure. This is often not the case, with CTCs used because of clinical need as opposed to patient preference.

Erm, to go through that whole procedure when maybe you could have had some other form of investigation and blood tests to eliminate that maybe it could just be piles … It's like a (sighs) a big thing to do without any other initial tests or investigation … Like a blood test or, er, an internal, a small internal, er, examination at the doctors or, I don't know, whatever tests they could do to eliminate certain things first.

(SPart03)

### 3.5 Control/Cure (*Beliefs About Bowel Screening and Control in Relation to Health Conditions, Including Coping With the Follow‐Up Procedure and Self‐Management in the Context of Health Conditions, e.g., Medications*)

Screening participants expressed few concerns about how their health conditions would impact their colonoscopy. Concerns were more generalised to potential uncertainty, discomfort and pain during the procedure. Those who considered themselves fit and healthy had even fewer concerns. Some participants were told by a screening nurse to stop or modify their medications before colonoscopy, with this viewed as an expected and normal part of the screening process for screening participants, particularly if they had prior experience managing their health conditions in the context of bowel screening.

… I do take er, Metformin, slow release, which makes me er, very constipated … I had to take er, Ex‐Lax laxatives, er, for seven days before taking the stuff to clear me bowel … I did this year. I did last year. So I know, I know exactly what's … there's no surprises for me, what I have to go, you know, what I have to do to get the bowel ready for the screening.

(SPart04)

When participants did have concerns about how their health conditions might impact colonoscopy, these were often mitigated by the SSP during telephone consultation. These consultations were described as helpful and reassuring. Some believed that, because their health conditions and age increased their likelihood of developing cancer, more regular tests should be offered; for example, more regular screening (including screening beyond age 74) or other tests in‐between screening, such as blood tests. Screening colonoscopists also praised the thorough job done by the SSPs, particularly data capture and preparing screening participants for successful completion of follow‐up procedures.

Screening staff frequently highlighted how it is common for some screening participants to not think about the onwards steps of bowel screening beyond sending back the stool test. In some instances, this was attributed to hoping for reassurance of a negative result, and in others, a lack of understanding or awareness of the full bowel screening pathway. This was described as particularly prevalent in certain groups, including those in care homes or with mobility issues. Screening staff described how it often takes a discussion between the screening participant and screening staff for the screening participant to understand what follow‐up involves.

We get lots and lots of participants who live in care homes. But they don't think of the ongoing progression of them … coming back with a positive, when some of these people are bed‐bound. They're never going to be able to withstand a procedure, or even go into the hospital for a meeting, you know? So, the challenge we face with that is that there's such a high turnover in care homes. So, we may go out and speak to people in care homes, but the staff is continually changing.

(HProf7)

When screening participants reflected on their knowledge and understanding of bowel screening, many understood that the purpose is to identify and treat cancer, with particular importance placed on early detection. Generally, screening participants were keen to have a follow‐up (e.g., colonoscopy) as they wanted to understand what was going on and treat any potential issues as soon as possible. Decisions to attend follow‐up were influenced by several factors, including losing loved ones to bowel cancer and weighing up screening risks and benefits, such as bowel perforation. Some also discussed the possible risks and benefits with their GP.

Erm and as I understand it, having the bowel perforated could be erm a sort of life changing outcome, erm so yeah, that was I suppose the one I wanted to, to weigh up carefully but I did come to the conclusion that you know, it was worth it, the benefits did outweigh erm possible outcomes … I did discuss, you know, the various pros and cons briefly with the GP and erm essentially, it was confirming the decision I'd come to basically that you know, yeah, benefits outweigh any risks.

(SPart11)

### 3.6 Suggested Improvements to the Screening Programme

Sharing of best practices and increased communication across Health Boards and specialities were widely acknowledged as approaches that could benefit both screening staff and participants. Improvements in communication were also a common theme for screening participants. Some screening participants with more complex screening journeys experienced miscommunication or a lack of communication between different appointments, making their screening journey confusing and stressful. Other participants reflected more generally on poor communication between different health services, particularly with their GP, and the frustrations caused by a lack of holistic care.

But there doesn't seem to be a lot of correspondence between the GP, the hospital, where I had my camera, er, and fluid up here, in [North Wales] … I just recently had another [letter], saying ‘do you want to have another [colonoscopy]?’ But I haven't had another test yet, to see whether I'm still bleeding or not.

(SPart17)

Screening participants were generally happy with the information provided in the screening pack; however, some reported that the information could be clearer and more concise. Suggestions included separating the Welsh and English language versions into different booklets, colour‐coded envelopes and forms, larger font, a cover letter outlining the contents of the pack and separating key information from additional information to aid navigation. Screening staff suggested that packs could signpost family members or carers to other sources of information or the screening helpline.

## 4 Discussion

### 4.1 Principal Findings

Areas of congruence and incongruence between screening staff and participants in their perceptions of the screening and diagnostic process have been identified in the current study. Whilst health professionals placed great emphasis on *identity* in the form of health conditions for their clinical decision‐making, there was a lack of screening participant awareness of the impact of health conditions on bowel screening. Comorbidities impact individuals differently, and subsequently, screening steps beyond the positive stool test need to be personalised. Therefore, despite being a population screening programme, bowel screening staff seek to individualise the service to meet participant needs. Time, resources and expertise are given over to finding out about an individual's health (*identity*) to make clinical recommendations that best suit the individual. However, screening participants are seemingly unaware of this focus. Screening participants with comorbidities may have a longer screening journey (*timeline*) due to the time taken to assess and gather information on, and plan for, the potential impact of their health conditions on the screening pathway. Consideration and use of alternative routes (e.g., investigations) can further add to the different screening experiences and *timelines* for those with health conditions. Interviews with screening participants identified that extended *timelines* can lead to heightened levels of worry and concern. A lack of awareness of potential reasons for extended timelines could contribute to this worry and concern, warranting further exploration in future research.

Comorbidities could mask the *cause* and attribution of potential symptoms of bowel cancer. For example, the presence of blood in stools could be attributed to comorbidities, leading to potentially symptomatic people being (inappropriately) in the screening service. The present study also explored how comorbidities can influence beliefs about follow‐up procedures. Screening participants who attribute their positive stool test to their comorbidity, for example, colitis or haemorrhoids, placed less importance on the need for further investigation and follow‐up. These potential masking effects and misattribution of symptoms highlight a possible gap in education and awareness of screening participants. It also highlights a potential area of focus for discussions in consultations and training for SSPs.

### 4.2 Strengths/Weaknesses of This Study

Embedding findings within the Common Sense Model [15] provides a theoretical framework for exploring findings. This study explores perceptions from both the screening participants themselves and the health professionals who consult with them, enabling a fuller picture to be developed and reflections to be made based on comparisons. For example, a possible disconnect is identified between the screening staff and participants when it comes to the focus given to health conditions. This disconnect could either reflect a genuine lack of impact of the health conditions experienced by the current screening participant sample or a lack of awareness about the actual impact of their conditions (*identity*) on bowel screening. Many screening participants in this study had not considered the relationship between their health conditions and bowel screening until asked during interviews, even for conditions that impact day‐to‐day life. This could reflect high public enthusiasm for cancer screening, with this previously shown to overshadow potential limitations or harms of screening [20].

Screening staff in this study represented all screening regions and job roles within Bowel Screening Wales. Despite efforts to recruit from across all of Wales for the screening participant interviews, interviewees predominantly came from North and West Wales. Future work including screening participants in areas not well represented here, including the most deprived areas, is needed to gain a full national representation and exploration of health inequalities [5, 9, 10].

### 4.3 Context of Other Literature

This research highlights the importance of recognising the variety of conditions encompassed within the multimorbidity label, including mental health, capacity issues and learning disabilities. Services therefore must not purely focus on physical conditions (e.g., chronic obstructive pulmonary disease). This study is the first to explore the key role of the SSP within the bowel screening service from the staff and screening participants' perspectives. Information gathering and the role provided by the SSP is key to starting to understand individual screening participant needs (*identity*). International research suggests that patient navigators (specialised healthcare workers who identify barriers for patients and ways to overcome them) can improve rates of bowel screening uptake and completion of colonoscopy following a positive stool test [21, 22, 23]. The current research suggests that holistic, person‐centred care is evident in bowel screening in Wales, particularly in the SSP role. The SSP role could be formalised to a more ‘Navigational’ role via formal training that focuses on further improving experience and informed decisions.

Low levels of screening participant tolerance for bowel preparation led to suggestions for alternate methods of screening, such as blood tests. Research into alternative technologies for screening, such as multicancer early detection tests [24, 25] and the acceptability of new technologies for bowel cancer (including blood tests and pill‐cameras) [26] is ongoing elsewhere. These research areas should ensure the representation of people with a variety of health conditions.

Perceived ability to complete and tolerate bowel preparation is used by screening staff to informally assess suitability for follow‐up (*consequences*). Whilst negative connotations of bowel preparation are well documented [27, 28], the current study adds to this literature with the highly emotive reaction towards this crucial part of bowel screening. Screening staff might want to reconsider thresholds for withstanding bowel preparation (consequences) and provide improved information (*control*).

### 4.4 Implications for Policy and Practice

Establishing a forum or meeting for screening staff to share experiences and best practices relating to comorbidity management could aid learning and information exchange, as well as reduce potential regional variations. Clearer communication between services and screening participants could ease the screening process, particularly for those with several comorbidities. This research has implications for informed screening decisions. Additional information or support could help increase *control* and alleviate concerns highlighted here by screening participants about coming to the end of their screening eligibility and the importance they place on early detection of bowel cancer. Education for both screening staff and participants could also be warranted to help reduce the possible disconnect regarding the screening and diagnostic process. The beliefs held by screening participants regarding the importance of screening compliance, early detection and timely treatment could represent positive reflections of screening uptake messaging, but could also be masking the realities of the screening process, particularly the complexities associated with comorbidities. Future work could consider implications for informed nonparticipation in screening and equitable and informed decisions along the bowel screening pathway [29, 30, 31].

### 4.5 Future Research

Quantitative research that explores the impact of comorbidity on the bowel screening pathway is needed to complement this qualitative study. The current study focused on any comorbidity. Future focus could be given to certain vulnerable screening participant groups; for example, care home residents, those with capacity issues or those who require assistance to complete the initial stool sample. Future work could also explore and try to disentangle general anxiety associated with wait times, particularly anxiety associated with delays while comorbid conditions are being managed, to optimise subsequent screening procedures and safety.

## 5 Conclusions

Early identification of comorbidities and associated complexities, followed by mitigating potential delays, is an important part of the bowel screening programme pathway. Screening staff place emphasis on health conditions in the clinical decision‐making process; however, screening participants have low awareness of its impact on bowel screening. Screening staff also focus on principles of doing no harm, and only recommending clinical investigations that are warranted based on the individual screening participant. This often contradicts the expectations of screening participants and their understanding of the purpose of screening, which focuses on the importance of early detection and subsequent treatment.

## Author Contributions

**Delyth Price**: writing–original draft, writing–review and editing, data curation, investigation, validation. **Katherine Brain**: supervision. **Sunil Dolwani**: supervision. **Adrian Edwards**: supervision. **Kerenza Hood**: supervision. **Stephanie Smits**: writing–original draft, writing–review and editing, conceptualisation, methodology, data curation, investigation, funding acquisition.

## Ethics Statement

Ethical approval for both qualitative studies was granted by the London Camden and Kings Cross Research Ethics Committee (reference: 18/LO/2212).

## Consent

Informed consent was received from all participants.

## Conflicts of Interest

The authors declare no conflicts of interest.

## Supporting information

Supporting information.

Supporting information.

Supporting information.

## Data Availability

The data that support the findings of this study are available from the corresponding author upon reasonable request.
