# Supplementary material for: Perceptions of the Impact of Comorbidity on the Bowel Cancer Screening Programme: Qualitative Study With Bowel Screening Participants and Staff
Source: Health Expect. 2024 Jul 2;27(4):e14126. doi: 10.1111/hex.14126 (PMC11217598; doi:10.1111/hex.14126)
Supplement: Supplementary file 1 — Supporting information. [file HEX-27-e14126-s001.docx]

# **Appendix 1: Screening participant interview topic guide**

**Interview prologue**

- Introduce the researcher, ensure the participant is comfortable; explain purpose of the interview; will take 30-45 minutes
- Check understanding; give opportunity for questions
- Explain that interview will be recorded.

1. **Illness representation & multi-morbidity (participant background)**

What do you know about bowel screening?

*Now going to ask you some questions about your general health:*

How would you describe your health in general? Have you ever been told by a health professional that you have a health condition?

- Probes: what impact does it have on you doing things day-to-day? how well do you manage your health conditions?
- Probes (no health conds): how is your general mobility? how is your general fitness? do you take any regular medication? how would you describe your health compared to most people you age?

*Thinking specifically about your bowel screening experience now:*

Have you talked about your general health with anyone working in the screening service? How did you feel when you were talking about your health with them?

- Probe: how confident did you feel? What reaction did you get?

How does your general health/ health condition(s) influence what you think about bowel screening?

1. **Barriers and facilitators to follow up tests**

What do you think are the benefits of taking part in bowel screening?

What do you think are the disadvantages of taking part in bowel screening?

- Probe: how does your general health influence these thoughts?

When you were told about the follow-up test (colonoscopy), what were your initial thoughts?

- Probe: how did your current level of health influence these thoughts? What other things did you think about; e.g. your age, mobility, medications, general fitness?

What helped you decide to have the follow-up test? How did your current health influence this decision?

- Probes: perceived positives to follow-up test, thoughts about not having follow-up

Did you talk to anyone else to help you make your decision? E.g. friends, family, GP

How would you describe your experience of the telephone consultation with the Specialist Screening Practitioner?

- What questions about your health were you asked?
- What did the screening nurse say when you mentioned your health condition? What discussions were had about your health condition and the next steps in the screening programme? Any mitigations
- How easy did you find it to answer these questions?
- Were you asked to change anything before the follow-up appointment? E.g. medications, diet – How did you feel about this?

What questions did you have about the follow-up test? Who answered them? How could this have been improved?

- Probe (if had procedure before): how did you feel about having another one? How do you feel this compared to last time?

1. **Follow-up experience**

*Thinking specifically about your colonoscopy now…*

When you went to the follow-up appointment, did you mention your current level of health?

- If yes: when was this? Why did you mention it at this point? Probe: how did the health professional respond to this?
- If no: why was this? Probe: if you had the procedure again, would you mention your health? (ask about specific health conditions listed on consent form/ in interview)

How did you think your health condition would influence your experience of the follow-up?

- Probe: mobility concerns, general fitness, medications, age
- If no: hypothetically?

When you had the follow-up test, did this expectation come true?

- Probes: did this expectation come true? Did your health condition/ general health make things harder/ more complicated, easier? Why was that?

What did you think the preparation would be like? What was it actually like?

- Probe: did you think your health would influence this? How did it?
- Probe: would further information have been helpful? What/ how?
- Probe: do you think HCPs are aware of how bad patients find the prep? Should they be made more aware?

What did you think the recovery would be like? What was it actually like?

- Probe: did you think your health would influence this? How did it?

Did you take anyone with you to the follow-up test?

- Probe: who and what support did they provide? what did they say to you?

Did anyone else help you at any other points in your screening journey? If so, who?

- Probes: with understanding info; aspects of bowel prep; consultations

How long a wait did you have for the follow-up test? How did you find this/ how did it make you feel? Was the reason for the delay explained to you?

Now you’ve had the follow-up test, what are the next steps for you?

Was your current level of health mentioned in these discussions? How do you feel your health conditions will impact these next steps for you?

- Probe: how do you feel about the next steps?

*Now going to ask a few questions about Covid:*

What impact did COVID have on your bowel screening experience?

- Probe any changes to processes (eg not allowed partner): how did that make you feel?

Did you talk about COVID with any of the healthcare professionals in the screening programme?

What impact did COVID have on how you think about your health?

1. **Improvements**

How can bowel screening be improved for people with health conditions?

- Probes: what should be focussed on? What support should be given?

What could be done do help health professionals who see people with health conditions in the screening programme?

How can bowel screening information be improved?

- Probe: what should there be more/ less information on?
- Probe: what about the initial screening invite pack? The follow-up test information?

How should this information be provided?

- Probes: would you like to receive the information in a different way/ format?

How could the interactions you’ve had with different healthcare professionals in the screening service be improved?

Is there anything else you’d like to mention about your experience of bowel screening?

*end recording*

- Thank participant for their time & contribution
- Check if they have any final questions
