# Supplementary material for: Perceptions of the Impact of Comorbidity on the Bowel Cancer Screening Programme: Qualitative Study With Bowel Screening Participants and Staff
Source: Health Expect. 2024 Jul 2;27(4):e14126. doi: 10.1111/hex.14126 (PMC11217598; doi:10.1111/hex.14126)
Supplement: Supplementary file 2 — Supporting information. [file HEX-27-e14126-s002.docx]

# **Appendix 2: Screening staff interview topic guide**

1. **Impact of multi-morbidity**

- Could you briefly explain what role you play in the bowel screening service?
- What impact do you think multi-morbidity has on bowel screening?
  - How does bowel screening currently accommodate people with multi-morbidity?
  - Prompts: how does that impact the service? How does that impact the individual patient?
- How do your consultations/clinical practice change if the patient has multi-morbidity? How does severity of multi-morbidity influence this?
  - What information do you use to help make clinical decisions for people with multi-morbidity? What factors would help you make this clinical recommendation/referral?
  - Is there anything else that you consider when making clinical recommendations/referrals for people with multi-morbidity? Probe e.g procedure invasiveness, prep, recovery?
- How do you communicate clinical recommendations/referral decisions to patients?
  - How does this differ for different types of multi-morbidity? How does this differ to patients without multi-morbidity?
  - What do you use to help you communicate? What could help you improve this communication?

1. **Illness representations and improvements**

- When you offer patients a clinical recommendation/referral, how does multi-morbidity influence their acceptance of it?
  - Why do you think this is?
  - What patterns do you see for certain types of multi-morbidity? What patterns do you see for different types of multi-morbidity severity?
  - What patterns do you see for gender/age/socioeconomic background +/-health literacy?
  - What common questions are you asked by patients or their families?
- What other barriers to acceptance of clinical recommendations/referrals do you see?
  - How do you try to overcome these? What could be done to reduce these barriers?
- How can the interaction you have with bowel screening participants with multi-morbidity be improved? (Probe: What would it take for this to happen?)
  - What could be done to help you as someone who sees people with multi-morbidity as part of the screening programme?
- How can bowel screening be improved for people with multi-morbidity?
  - What should be focused on? Should more or less of anything be done? What about the information people with MM receive?
  - What support should be given? When? How should it be given?
  - How can communication in bowel screening be improved for people with MM? (Probe: Who should the communication be improved between? Screening service and participant? Participant and health professional? What benefit do you think this would bring?)
  - If screening was based on risk-stratification, how receptive do you think patients with MM and their families would be to doing less bowel screening?

1. **Covid-19**

- What impact do you think Covid-19 had on bowel screening for people with multi-morbidity?
- What impact did multi-morbidity have on your practice during the Covid-19 pandemic? How did your decision-making change during this time?
  - How did your clinical recommendations/referrals change during this time?
  - What risk stratification and prioritisation processes did you follow?
  - Were there any other practicalities in the context of patient multi-morbidity?
- What impact did Covid-19 have on acceptance of clinical recommendations/referrals for people with multi-morbidity?
  - What impact did Covid-19 have on patients’ decision-making? What questions did patients with multi-morbidity tend to have?

**Other general probes that could be used:**

- Why do you think that might be important?
- Why do you think that should be changed?
- What could be done to improve that?
- Can you tell more about that?
- Is there anything else you would like to mention about your experience with patients with multi-morbidity in bowel screening?

At the end of the interview the researcher will thank the participant for their contribution The researcher will answer any questions the participant has and thank them again for their time.
