# Supplementary material for: Perceptions of the Impact of Comorbidity on the Bowel Cancer Screening Programme: Qualitative Study With Bowel Screening Participants and Staff
Source: Health Expect. 2024 Jul 2;27(4):e14126. doi: 10.1111/hex.14126 (PMC11217598; doi:10.1111/hex.14126)
Supplement: Supplementary file 3 — Supporting information. [file HEX-27-e14126-s003.docx]

Supplementary File 1. Screening Participant Characteristics

| **Participant** | Age | Gender | Conditions | Total number conditions | SCQ score | WIMD rank |
| --- | --- | --- | --- | --- | --- | --- |
| P01 | 64 | F | High blood pressure, depression, osteoarthritis/ degenerative arthritis | 3 | 6 | 3 |
| P02 | 66 | F | None | 0 | 0 | 2 |
| P03 | 59 | F | None | 0 | 0 | 5 |
| P04 | 70 | m | High blood pressure, diabetes, liver disease, rheumatoid arthritis | 4 | 6 | 4 |
| P05 | 66 | m | None | 0 | 0 | 5 |
| P06 | 70 | m | Heart disease, high blood pressure, osteoarthritis/ degenerative arthritis | 3 | 5 | 5 |
| P07 | 58 | m | High blood pressure | 1 | 2 | 5 |
| P08 | 69 | f | Back pain | 1 | 1 | 2 |
| P09 | 58 | m | None | 0 | 0 | 5 |
| P10 | 62 | f | High blood pressure, diabetes, liver disease, anaemia or other blood disease, back pain, rheumatoid arthritis | 6 | 15 | 3 |
| P11 | 69 | m | Back pain | 1 | 1 | 3 |
| P12 | 59 | m | None | 0 | 0 | 4 |
| P13 | 58 | m | Anaemia or other blood disease | 1 | 2 | 3 |
| P14 | 66 | f | Heart disease, high blood pressure, lung disease, depression | 4 | 10 | 4 |
| P15 | 74 | m | Diabetes | 1 | 2 | 4 |
| P16 | 71 | m | Heart disease, high blood pressure, diabetes, cancer, osteoarthritis/ degenerative arthritis, back pain | 6 | 12 | 4 |
| P17 | 69 | f | Heart disease, ulcer or stomach disease | 2 | 4 | 5 |
| P18 | 68 | f | Anaemia or other blood disease | 1 | 1 | 5 |
| P19 | 65 | f | High blood pressure, diabetes, kidney disease, liver disease, anaemia or other blood disease, depression, rheumatoid arthritis | 7 | 13 | 2 |
|  |  |  |  | Avg = 2.1 | Avg = 4.2 |  |
